# Mobility Screening for Fall Risk Assessment in a Population-Based Sample of Older Adults: An Analysis From the CLSA

**DOI:** 10.1093/geroni/igaa057.860

**Published:** 2020-12-16

**Authors:** Marla Beauchamp, Ayse Kuspinar, Nazmul Sohel, Alexandra Mayhew, Lauren Griffith, Parminder Raina

**Affiliations:** McMaster University, Hamilton, Ontario, Canada

## Abstract

Existing guidelines for fall prevention in older adults recommend mobility screening for fall risk assessment; however, there is no consensus on which test to use and at what cut-off. This study aimed to determine the accuracy and optimal cut-off values of commonly used mobility tests for predicting falls in the Canadian Longitudinal Study on Aging (CLSA). Mobility tests at baseline included the Timed Up and Go (TUG), Single Leg Stance (SLS), chair-rise, and gait speed test. Inclusion criteria were: age ≥ 65 years and history of a fall or mobility problem at baseline. Accuracy of fall prediction at 18-months for each mobility test was measured by the area under the receiver operating curve (AUC). Of 1,121 participants that met inclusion criteria (mean age 75.2 ± 5.9 years; 66.6% women), 218 (19.4%) participants reported ≥1 fall at 18-months. None of the mobility tests achieved acceptable accuracy for identifying individuals with ≥1 fall at follow-up. Among women 65-74 and 75-85 years, the TUG identified recurrent fallers (≥2 falls) with optimal cut-off scores of 14.1 and 12.9 seconds (both AUCs 0.70), respectively. Among men 65-74 years, only the SLS showed acceptable accuracy (AUC 0.85) for identifying recurrent fallers with an optimal cut-off of 3.6 seconds. Our findings indicate that for a population-based sample of community-dwelling older adults, commonly used mobility tests do not have sufficient accuracy for identifying fallers. The TUG and SLS can identify older adults at risk for recurrent falls, however their accuracy and cut-off values vary by age and sex.

